# AudBility: test-retest reliability in typically developing children aged 6 to 7 years

**DOI:** 10.1590/2317-1782/20212021219en

**Published:** 2022-07-25

**Authors:** Tamy Nathalia Tanaka, Nádia Giulian de Carvalho, Maria Francisca Colella-Santos, Maria Isabel Ramos do Amaral

**Affiliations:** 1 Programa de Pós-graduação em Saúde, Interdisciplinaridade e Reabilitação, Universidade Estadual de Campinas – UNICAMP - Campinas (SP), Brasil.; 2 Programa de Pós-graduação em Saúde da Criança e do Adolescente, Centro de Investigação em Pediatria, Universidade Estadual de Campinas – UNICAMP - Campinas (SP), Brasil.; 3 Departamento de Desenvolvimento Humano e Reabilitação, Faculdade de Ciências Médicas – FCM, Universidade Estadual de Campinas – UNICAMP - Campinas (SP), Brasil.

**Keywords:** Screening, Child, Data Accuracy, Validation Study, Auditory Perception

## Abstract

**Purpose:**

to investigate the test-retest reliability of the AudBility program in typically developing children aged six-seven years.

**Methods:**

29 children, male and female, right-handed, native Portuguese speakers and adequate school performance for the age group studied, underwent previous meatoscopy, immittance measurements and the AudBility program was applied, composed of a self-perception questionnaire and auditory tasks, being analyzed the abilities of sound localization, auditory closure, figure-ground, dichotic digits test, temporal resolution, and temporal ordering of frequency and duration. The program was designed and reapplied with an interval of one week under the same conditions. The performance in each task was presented from central tendency and dispersion data and was conducted using the Intraclass Correlation Coefficient (ICC), based on the 95% confidence interval (CI).

**Results:**

The analyses showed a positive and significant ICC (p<0.01) for the questionnaire and auditory tasks, except for auditory closure, in the right and left ears and figure-ground in the left ear. The questionnaire mean ICC was 0.742 and ranged from −0.012 to 0.698 for the auditory tasks.

**Conclusion:**

Based on mean results and upper limit of the CI, the findings showed agreement between moments, classified as good for the questionnaire and moderate to good for five of the seven auditory analyzed tasks (ICC>0.05 and <0.9). The results of the reliability study represent an important parameter for validating the program for the studied age group.

## INTRODUCTION

Central auditory processing (CAP) is related to the efficiency of auditory information perception by the central auditory nervous system (CANS) and the underlying neurobiological activity, constituting a group of specific skills that an individual must have to understand what is heard^(1)^. These skills involve different auditory mechanisms, such as sound localization and lateralization, discrimination and recognition of auditory patterns, dichotic listening, listening in environments with competitive sounds or degraded acoustic signal^(1)^.

To assess CAP abilities, it is important to understand the relationship between the CANS and the peripheral auditory nervous system (PANS). The PANS comprises the outer, middle, and inner ears and the auditory nerve. It is responsible for capturing and transmitting the auditory signals to the CANS, which will interpret the information. Then, these are distinct but interdependent processes. CAP assessment can be performed either through electrophysiological procedures or a battery of special behavioral tests, which identify the presence of central auditory processing disorder (CAPD), that is, a deficit in the neural processing of auditory stimuli and consequent impairment of the assessed auditory skills^(1)^.

Studies show peripheral auditory dysfunction—with or without a history of secretory otitis media in early childhood—can result in immature auditory pathways and central auditory skills^(2,3)^. In addition, alterations in auditory skills can affect a child’s learning and development processes, due to an interdependence with higher cognitive functions, such as language, memory, and attention^(4,5)^. CAPD in school-age children can coexist with other disorders, such as attention deficit hyperactivity disorder (ADHD), language development disorders, dyslexia, among others^(6,7)^. However, specific signs of CAPD related to deficits along the auditory pathway can also occur in isolation, such as problems to understand speech in noisy environments, poor understanding in situations with increased speech rate, inability to discriminate similar words or sounds or conduct complex auditory commands^(8)^. These complaints lead to issues that, in most cases, are seen in a low academic performance^(9)^. Studies show an association between the presence of CAPD and specific learning disorders^(5)^, mainly related to reading and writing processes and mathematical calculations, as well as phonological deficits^(10)^ and speech perception, including suprasegmental aspects of speech^(11)^.

Auditory screening, which includes procedures that evaluate both the peripheral and central segments, allows an early identification and immediate intervention of auditory alterations in this population, minimizing potential school losses^(12)^. In recent years, the development of new screening test batteries for auditory skills has been the topic of studies^(13-15)^ aiming to combine the countless possibilities of technology in health with procedures that fulfill the screening requirements, considering the easy access and application time, different auditory mechanisms, and interactive activities^(15)^.

Considering the above, sensitivity, specificity and reliability data must be studied to support the validation of new procedures. In this context, the “AudBility” battery was created in Brazil. It is an online playful and interactive program for screening auditory skills that has been studied and improved^(15)^. The program consists of auditory tasks and a self-perception questionnaire for children, parents, and teachers. In order to continue AudBility-related studies and contribute to program validation, this study aimed to investigate the test-retest reliability in a group of typically developing children aged six to seven years.

## METHODS

This is a cross-sectional, descriptive-analytical, quantitative reliability study approved by the Research Ethics Committee of the State University of Campinas (Unicamp) under approval n.º 1.561.422 and conducted in partnership with a public school. The parents and/or guardians of all children included in the study signed an informed consent form (ICF) and an assent form.

### Selection of subjects and data collection

First, as part of the study of normative data from AudBility, which includes our present investigation^(15)^, invitation letters were sent to parents/guardians of male and female children, aged 6 to 8 years, native Portuguese speakers. Children with a confirmed diagnosis of peripheral hearing loss were excluded, as well as those with cognitive/neurological alterations that affected their neuropsychomotor or language development that were known to the pedagogical coordination or the child’s teacher or included in school records – conditions that could affect the child’s understanding of the tasks to be performed.

**Figure 1:**
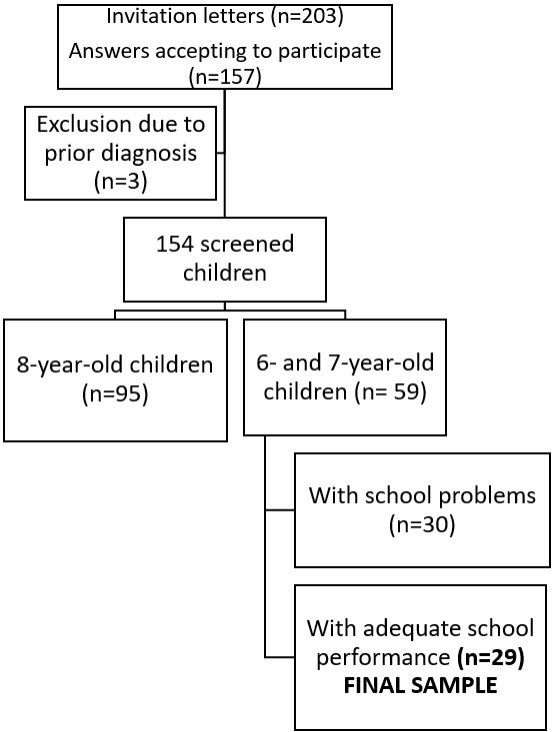
Selection criteria of final study sample.

In total, 203 invitation letters were sent. Of these, 157 (77%) agreed to participate. Three children did not meet the selection criteria and were excluded, totaling 154 children who underwent peripheral screening and AudBility procedures. Of all 154 children, 95 were 8 years old and had already been screened more than two months before, which did not allow the inclusion of 8-year-old children in the reliability analysis. In addition, this study included only children with proper school performance for the age group according to the teacher in charge, without retention or additional school tutoring, and who agreed to participate in the retest stage. Therefore, the final sample of this study consisted of 29 children aged 6 and 7 years, 16 (55.17%) female and 13 (44.83%) male children, mean age 6.68 years (±0.54). Figure 1 illustrates the selection process of the final sample.

Regarding the procedures, before applying the AudBility battery, meatoscopy was performed with a Heine otoscope and immittance measurements using Interacoustics MT10 to assess the middle ear conditions. Children who had excess cerumen were referred to removal service and later included for data collection. Children who presented altered results in immittance measurements, that is, type B or C tympanogram, and absence of ipsilateral acoustic reflex, were referred to complete audiological evaluation, otorhinolaryngological evaluation, and were excluded from data analysis.

After immittance screening, the AudBility program was applied in a quiet room provided by the school, away from the other classrooms and schoolyard. During breaks and changes in school shifts, the application was interrupted by the researcher. The battery was applied with the help of a speech therapist with experience in the AudBility program, who was also one of the researchers of this study. A desktop computer connected to the internet and Panasonic RPHC720 noise-canceling over-ear headphones were used.

AudBility consists of a self-perception questionnaire about auditory skills and auditory tasks. In addition, it has three modules describing the tasks, one for children aged 6 to 8 years; one for children aged 9 to 12, provided they are able to read; and one for children over 13 years old. This study analyzed data collected from the application of the module to 6- to 8-year-old children. In this module, the activities do not require the student to know how to read and have pictographic resources.

The questionnaire assessing the self-perception of skills – an adapted version inserted in the program^(16)^ – has 12 questions to be answered with the frequency of occurrence of everyday situations, with pictographic support for the child’s answers and a 5-point Likert scale with the following options: always (1.0), often (2.0), sometimes (3.0), rarely (4.0), never (5.0). Every question is preceded by an example of situation to facilitate the child’s understanding.

The researcher read the explanation screen of the questionnaire from the program and, after ensuring the child understood it, he assisted in questionnaire application by reading the questions aloud and helping the child check the answer on the computer screen. At the end, the program computed the score for individual questions and calculated the total score, ranging from 12 to 60. Chart 1 describes all 12 questions.

**Chart 1:** Description of self-perception questionnaire used in the study.

**Questions**	**Example of situation / Question**
**Answer options**	**Always / Often / Sometimes / Rarely / Never**
1	You are in the classroom or in a place where people are talking, / **Is it difficult for you to hear or understand what the teacher is saying?**
2	The teacher or another person is talking too fast to you, / **Is it difficult for you to understand what the teacher is saying?**
3	The teacher or another person is giving oral instructions (explanations) to you, / **Is it difficult for you to follow oral instructions?**
4	The teacher or another person is talking to you in a quiet environment, / **Is it difficult for you to clearly hear and understand without changing any letter?**
5	When the teacher or a friend is talking to you, / **Do you feel that sometimes you hear well and sometimes you don’t?**
6	You are in the classroom or schoolyard and someone calls your name, / **Is it difficult for you to find out where the sound is coming from?**
7	The teacher or another person is talking to you, / **Do you ask him or her to repeat what he or she said?**
8	You are in the classroom, / **Are you easily distracted?**
9	Last year at school, / **Did you have difficulties learning?**
10	Are you doing an activity, / **Is it difficult for you to pay attention?**
11	When you are in the classroom or at home, / **Do people say you are daydreaming or inattentive?**
12	When you are at school or at home, / **Are you disorganized?**

For the auditory tasks, the program automatically calibrated the sound output and the headphones, with the computer volume mixer set to 50%. The researcher asked the child if that was a comfortable listening level. The volume could be adjusted as required.

The screening protocol consisted of seven auditory tasks, as follows: sound localization, figure-ground, auditory closure, dichotic digits test, temporal resolution, and temporal ordering of frequency and duration. As the auditory closure and figure-ground tasks were analyzed by ear, there were nine analysis variables. The tasks were interactive and playful and were designed considering behavioral tests that are part of the diagnostic battery, but with different acoustic parameters and a reduced amount of stimuli in every task. Chart 2 describes all tasks and the scoring method.

**Chart 2:** Description of skills and parameters related to each task comprising the screening protocol (total: 7 tasks).

**AudBility task**	**Parameters**
Sound localization	10 target situations: right, left or above/behind;
	10 presentations – each error: 10%
Binaural integration (dichotic digits test)	4 numbers presented concurrently (two in the right ear and two in the left ear).
	20 digits per ear – each error: 5% (answer analyzed considering total correct answers obtained from RE+LE).
Figure-ground (monoaural)	10 sequences per ear in which the child hears a story and, at the same time, a sentence related to a picture and must point to the **picture**.
	Each error is equivalent to 10%.
Auditory closure (monoaural)	10 sequences per ear in which the child hears an acoustically modified word and must recognize the word among the **pictures** displayed on the screen.
	Each error is equivalent to 10%.
Temporal resolution	Simple 1000 Hz stimulus (whistle) with intervals (gaps) of 20 ms, 15 ms, 10 ms, 6 ms, 4 ms, and 0 ms between them. In each presentation, the child hears a sequence of six sounds with random gaps and is instructed to count how many he/she can perceive/hear. Each gap appears 10 times.
	The threshold considered is the lowest the child perceives, at least 6 of 10 presentations or more.
Temporal ordering, duration	10 sequences of three combinations of **800 Hz/400 ms** (LONG-L) and **800 Hz/200 ms** (SHORT-S) pure tones. Silence time of **350 ms** (LLS, SSL, LSL, SLS, SLL, and LSS) between sequences.
	Each error is equivalent to 10%.
Temporal ordering, frequency	10 sequences of three combinations between pure tones: a **700 Hz** gross stimulus (GROSS-G) and a **1500 Hz** fine stimulus (FINE-F) of **350 ms** duration, such as GGF, FFG, FGF, GFG, GFF, and FGG.
	Each error is equivalent to 10%.

**Caption:** Hz = Hertz; ms = milliseconds; RE = right ear; LE = left ear

Every task has an explanation and training screen. The task only started after confirming the child understood the activity and the researcher helped the child answer on the computer screen. At the end of task, the program displayed a screen congratulating the child with the percentage of correct answers and/or threshold obtained. This information is stored in the program and can be displayed with a summary of the child’s performance in all the tasks and the questionnaire score (total and for every question). This screen also shows information about the total battery application time and time per task.

One week after the program application, all 29 schoolchildren were invited to participate in the retest stage. In this stage, the same evaluator reapplied the procedures of the test stage, including meatoscopy, immittance measurements, and the AudBility program. The same conditions of the test stage were present in terms of material resources (room, computer, and phone), parameters of stimulus intensity, and order of tasks, instructions for every task, and method of application/help with marking the results. The participants were equally motivated to show the same level of attention while performing the tasks in both moments of program application.

### Statistical analysis

For data analysis, the average performance for each of the nine variables after the application of the tasks was calculated using the percentage of correct answers or average threshold for the temporal resolution task. These data were presented as measurements of central tendency and dispersion, and as the mean score obtained in the self-perception questionnaire.

An intraclass correlation coefficient (ICC) was used to assess the agreement or disagreement between the test and retest stages, i.e., the degree of reliability, based on a mixed two-factor model of absolute agreement using single measurements^(17)^. In the analysis, negative ICC values are interpreted as disagreement and positive values as agreement between the stages. Zero indicates absence of disagreement or agreement between the stages (chance). In the ICC analysis, the correlation level should be evaluated, considering not only the mean ICC but also the 95% confidence interval, based on the following classification^(17)^: values below 0.5 indicate poor reliability, between 0.5 and 0.75 moderate reliability, between 0.75 and 0.9 good reliability, and values above 0.9 indicate excellent reliability.

## RESULTS

Table 1 shows the central tendency and dispersion measurements based on the answers to the questionnaire and the mean performance of correct answers in the auditory processing screening tasks obtained through the application of AudBility, for the two moments of program application.

**Table 1:** Sample characterization in relation to the results of the questionnaire and AudBility tasks for both times of application (n=29)

**Variable**	**Evaluation moment**	**Mean**	**SD**	**Median**	**Min.**	**Max.**
Questionnaire (score)	Test	45.72	7.46	46	30.00	56.00
		[42.87, 48.62]		[45.00, 49.50]		
	Retest	45.66	6.91	46,00	30.00	57.00
		[43.15, 48.24]		[42.00, 50.00]		
Localization (%)	Test	82.07	11.77	80	50.00	100.00
		[77.71, 86.40]		[80.00, 80.00]		
	Retest	90.69	12.23	100	60.00	100.00
		[85.83, 94.84]		[100, 100]		
Digits – general (%)	Test	82.84	8.47	85	67.50	97.50
		[79.67, 85.89]		[81.25, 85.00]		
	Retest	88.71	8.25	92.5	67.50	100.00
		[85.69, 91.59]		[90.00, 92.50]		
Auditory closure – RE (%)	Test	89.66	12.10	90	60.00	100.00
		[85.00, 93.93]		[90.00, 90.00]		
	Retest	97.59	5.11	100	80.00	100.00
		[95.67, 99.31]		[100, 100]		
Auditory closure – LE (%)	Test	87.24	16.23	90	30.00	100.00
		[80.39, 92.74]		[90.00, 90.00]		
	Retest	96.21	6.22	100	80.00	100.00
		[93.79, 98.33]		[100, 100]		
Total figure-ground – RE (%)	Test	84.14	11.50	90	50.00	100.00
		[79.25, 88.13]		[90.00, 90.00]		
	Retest	87.24	8.41	90	70.00	100.00
		[84.17, 90.34]		[90.00, 90.00]		
Total figure-ground – LE (%)	Test	81.03	17.18	80	30.00	100.00
		[73.47, 87.14]		[80.00, 90.00]		
	Retest	88.97	9.00	90	70.00	100.00
		[85.76, 92.00]		[90.00, 90.00]		
Temporal resolution (threshold – ms)	Test	5.38	4.21	4	4.00	20.00
		[4.10, 6.93]		[4.00, 4.00]		
	Retest	4.62	2.98	4	4.00	20.00
		[4.00, 5.85]		[4.00, 4.00]		
Temporal ordering, frequency – Total (%)	Test	68.28	21.39	70	20.00	90.00
		[60.46, 75.12]		[60.00, 80.00]		
	Retest	76.55	21.43	80	30.00	100.00
		[68.52, 83.45]		[80.00, 80.00]		
Temporal ordering, duration – Total (%)	Test	21.03	23.04	10	0.00	90.00
		[13.75, 29.66]		[10.00, 20.00]		
	Retest	26.21	20.94	20	0.00	90.00
		[19.10, 33.79]		[10.00, 30.00]		

**Caption:** ms = milliseconds; RE = right ear; LE = left ear; SD: standard deviation; Min.: Minimum; Max.: Maximum

Table 2 shows the test-retest reliability analysis for the self-perception questionnaire and auditory processing screening tasks from AudBility. The analyses showed positive ICC, that is, agreement between the application moments, with statistical significance in the questionnaire and six of the nine variables of the auditory tasks (p<0.05). Based on the mean result and the upper limit of the confidence interval (CI), the findings showed good agreement between the moments for the questionnaire, with moderate to good agreement for five of the nine auditory variables (ICC >0.05 and <0.9), and moderate to good agreement for the tasks of temporal ordering of duration, temporal resolution, dichotic digits test, and figure-ground in the right ear, and moderate for the task of temporal ordering of frequency. The task of sound localization showed agreement ranging from poor to moderate when considering the upper limit of the CI, and poor agreement was observed for the tasks of auditory closure, in the right and left ears, and figure-ground in the left ear.

**Table 2:** Test-retest reliability analysis of the questionnaire and auditory variables from the auditory tasks applied to the study sample (n=29)

**Screening**	**ICC**	**P**
Questionnaire	0.742	**< 0.001** ^*^
	[0.519, 0.871]	
Localization	0.253	0.046*
	[−0.064, 0.543]	
Digits – general	0.558	< 0.001*
	[0,052. 0.803]	
Auditory closure – RE	0.178	0.102
	[−0.107, 0.469]	
Auditory closure – LE	0.095	0.268
	[−0.188, 0.400]	
Total figure-ground – RE	0.589	**< 0.001** ^*^
	[0.296, 0.782]	
Total figure-ground – LE	−0.012	0.527
	[−0.317, 0.323]	
Temporal resolution	0,698	< 0.001*
	[0.455, 0.845]	
Temporal ordering, frequency – Total	0.51	0.001*
	[0.191, 0.733]	
Temporal ordering, duration – Total	0.554	0.001*
	[0.249, 0.761]	

* = Statistically significant value at the 5% level (p ≤0.05).

**Caption:** ICC = Intraclass correlation coefficient

## DISCUSSION

In a validation process, the reliability of the construct is related to the constancy, precision, and homogeneity of the results when the individual is evaluated more than once. It is, therefore, a highly important assessment for the significance of collected measurements. Different criteria are used in reliability studies, one of the most frequently used in the health area is the test-retest technique, in which the same measurement is applied twice to the same subjects and agreement is observed between the two moments^(18)^.

This technique can be affected by environmental factors such as evaluators, sample characteristics, instrument type, and administration methods. Therefore, the steps should be applied under the same conditions and analyzed in a statistically coherent manner^(19)^. Ideally, retests, performed a few days or a few weeks after the test, should present similar results^(20)^. The literature has studies assessing auditory processing with up to 60-day period between the two moments of analysis^(14,21,22)^. In screening studies, the sample rate varies from at least 5% to 10% of the total screened sample^(13,14)^. Our study attempted to respect this guidance, and reassessments were conducted one week later by the same evaluator, under the same evaluation conditions. Regarding the sample rate, considering all 154 children who underwent screening, the sample of 29 children (18.83%) was, therefore, a representative sample.

According to the descriptive data in Table 1, regarding the scores obtained in the self-perception questionnaire and the auditory tasks, the sample had an expected performance for typically developing children, when compared to data from a previous study assessing the initial application of AudBility in children without school problems but with typical development from an older age group^(15)^. In this previous study, the older age group with similar results reinforces the good performance of our sample, consisting of younger students. In addition, the mean score above 45 obtained in the questionnaire is also consistent with the expected performance described in a study conducted with the SAB questionnaire in its translated version^(23)^, reinforcing the absence of risk for CAPD in the sample, even considering it was selected with the limitation of a criterion based on a qualitative analysis of the teacher and school records, without a formal school performance assessment.

Different statistical tests can be used in the test-retest analysis, including the intraclass correlation coefficient (ICC), which is frequently recommended in recent literature, allowing a true analysis of agreement between moments. Other correlation measurements are Pearson’s correlation coefficient, Spearman’s correlation coefficient, paired Student’s t-test, and Wilcoxon’s corresponding non-parametric test^(17)^. This variability of analyses does not favor direct comparisons between different findings, despite being a frequent topic in the field. Over the last decades, many studies have assessed reliability using test-retest methods, including development of screening batteries or auditory processing tests^(13,14,19,24-27)^.

Good test-retest reliability of the self-perception questionnaire studied here highlights the importance of including this type of instrument as a complementary assessment of auditory performance in auditory screening battery at school. Several authors emphasize this fact considering the questionnaire is a simple and reliable method, as long as it is designed according to adequate psychometric principles and validation data are available to demonstrate the functional impact of CAPD on daily life, from a qualitative point of view^(23,28)^. The result also highlights the findings discussed in a previous study in which the same instrument effectively differentiated a group of students based on the variable of academic performance^(16)^, emphasizing the importance of validation studies that include not only test-retest reliability but also a study of sensitivity and specificity with cutoff point for CAPD risk and occurrence, based on a correlation with diagnostic tests^(13)^.

Despite the variability of studies with discrepant methods to obtain data and analyze the results, the findings regarding reliability of the auditory tasks are consistent with other studies. The first version of the SCAN-A (Test for Auditory Processing Disorders in Adolescents and Adults)^(27)^ was assessed using test-retest reliability with 38 participants, with an average of 40 days between the two moments of analysis. The findings were considered unsatisfactory, with poor correlations in most battery tasks. Later, other researchers^(24)^ studied the SCAN-C, a version for children, and found improved scores in the second application, suggesting the effect of practice. Also, a revised version of the battery was applied to a sample of 680 schoolchildren; 145 (21.3% of the total sample) were included in a test-retest analysis with a 1-week interval between applications, and low to moderate correlations were found, except for the figure-ground skill^(26)^, data that agree with our findings. In another validation study that analyzed a screening battery named STAP – Screening Test for Auditory Processing^(14)^, 141 schoolchildren were screened by the program and, of these, 50 (10% of the total sample) underwent the retest stage with a two-month interval between assessments. Pearson’s correlation coefficient showed statistically significant findings ranging from 0.82 to 0.93, suggesting good reliability.

More recently, in an initial validation study for Feather Squadron^(13)^, a computer screening program that is similar to AudBility, the viability of program application to different age groups was studied together with test-retest analysis with 1-week interval between the moments of application, involving 5 auditory mechanisms evaluated (sound localization and lateralization, recognition of auditory patterns, temporal aspects of hearing, auditory discrimination, and auditory performance with degraded sounds). Using the Wilcoxon signed-rank test, the authors found the effect of practice with a better performance in the second application in three of the five mechanisms evaluated. They also found the same effect in three conventional tests from the behavioral evaluation battery of auditory processing, showing a correlation between screening and diagnostic procedures, but not in all of them.

Then, the different studies discussed here present batteries with minimal agreement or moderate correlation in most tasks that comprise the CAP screening protocols, although not all tests demonstrate this expected degree of correlation^(13,14,24,27)^. Therefore, the findings of our study indicate reliability of the program applied to this age group, contributing to its validation.

Our findings did not demonstrate statistically significant results in the tasks of auditory closure in both ears and figure-ground in the left ear. Auditory closure is the ability to understand an incomplete or distorted sound message, which does not favor decoding phonemic aspects of a speech signal. Figure-ground skill consists in interpreting sentences in the presence of a competitive message. These are essential auditory skills for speech understanding, especially in the school environment^(29)^. When considering the description of the average performance of correct answers in the tasks, both for sound localization, whose agreement was low, and for auditory closure and figure-ground skills in the left ear, differences were observed between the moments, with improved performance in the retest when compared to the other tasks, with positive results and a higher degree of agreement, suggesting a possible effect of practice, although a paired statistical analysis of this difference was not performed. Even so, we may consider the hypothesis that such difference may explain disagreement between the moments for these tasks.

This discussion and analysis are relevant particularly for future studies that aim to use the AudBility battery in order to monitor the maturational development of auditory skills or as a monitoring instrument for CAPD rehabilitation in children. The authors emphasize the calculation of ‘difference – D’ that represents the degree of improvement or worsening between the moments must be considered and deducted to obtain the actual degree of improvement (or worsening) of the child over time, in a process of intervention or even for proper understanding of the instrument sensitivity for the measurement of neuromaturational development of auditory skills over time^(19)^.

Some factors must be discussed and may also support hypotheses regarding differences in performance between the evaluation moments, considering the tasks presenting low agreement. The fact that school performance was not evaluated with a validated formal instrument, but based on the teacher’s report, justifies a more heterogeneous sample from the point of view of learning processes. Although limiting, this aspect does not make the reliability study unfeasible, since the analyses are comparative different moments of the same subject. Despite the above, this aspect does not seem to fully explain the discrepancies found, since the performance was better in the second application.

Another aspect to be discussed is the influence that CANS-independent cognitive mechanisms can have on the expected performance of an individual undergoing CAP assessment, such as memory and attention^(30)^. Although presenting typical development, the absence of formal assessments that include cognitive and language screening favors a more heterogeneous sample, a factor that can also explain the disagreement between the evaluation moments in some tasks. However, it is a school screening application, and an extensive evaluation protocol would make the proposal unfeasible. In addition to cognitive aspects, behavioral factors or intrinsic variability of each individual must be considered, such as sleep, tiredness, alertness, appetite, and degree of motivation to perform activities. From a methodological perspective, although the technical conditions of program application were the same in both moments, it was not possible to control all aspects mentioned above. The child was not necessarily evaluated at the same time as the school period and may be more or less tired from one week to the next, for example. From a motivational point of view, familiarity with the technological resource may be an additional factor of motivation and engagement when performing the activities, which may also have contributed to a better performance in the second evaluation.

Finally, in general the mean ICC obtained in tasks with a significant positive result is equivalent to a moderate degree of reliability, but when considering the analysis based on the confidence interval, this classification ranged from bad to good and produced uncertainty data from a statistical perspective. As discussed above, although in agreement with other validated screening batteries, this finding justifies the importance of further studies assessing a larger sample of schoolchildren in order to limit the confidence interval obtained and investigate the psychometric properties of the tasks for other age groups and other pediatric populations, which could provide a better understanding of the program validation for different populations.

## CONCLUSION

Considering the age group studied and the results found, AudBility showed good agreement in the questionnaire between the evaluation times; moderate agreement in the tasks of temporal ordering of frequency and duration, temporal resolution, figure-ground in the right ear, and dichotic digits test; and low agreement, although statistically significant, in the sound localization task. The findings from the reliability study represent an important parameter for AudBility validation.
