# Supplementary material for: AudBility: test-retest reliability in typically developing children aged 6 to 7 years
Source: Codas. 2022 Jul 25;34(6):e20210219. doi: 10.1590/2317-1782/20212021219en (PMC9886299; doi:10.1590/2317-1782/20212021219en)
Supplement: Translation

# Translation: AudBility: confiabilidade teste-reteste em crianças com desenvolvimento típico de 6 e 7 anos

**DOI:** 10.1590/2317-1782/20212021219pt

**Authors:** Tamy Nathalia Tanaka, Nádia Giulian de Carvalho, Maria Francisca Colella-Santos, Maria Isabel Ramos do Amaral

**Affiliations:** 1 Programa de Pós-graduação em Saúde, Interdisciplinaridade e Reabilitação, Universidade Estadual de Campinas – UNICAMP - Campinas (SP), Brasil.; 2 Programa de Pós-graduação em Saúde da Criança e do Adolescente, Centro de Investigação em Pediatria, Universidade Estadual de Campinas – UNICAMP - Campinas (SP), Brasil.; 3 Departamento de Desenvolvimento Humano e Reabilitação, Faculdade de Ciências Médicas – FCM, Universidade Estadual de Campinas – UNICAMP - Campinas (SP), Brasil.

**Keywords:** Triagem, Criança, Confiabilidade dos Dados, Estudo de Validação, Percepção Auditiva

## Abstract

**Objetivo:**

investigar a confiabilidade teste-reteste do programa AudBility em crianças com desenvolvimento típico de seis a sete anos.

**Método:**

29 crianças, sexo masculino e feminino, destros, falantes nativos do Português e desempenho escolar adequado para a faixa etária estudada de acordo com o professor foram submetidas aos procedimentos de meatoscopia, imitanciometria e foi aplicado o programa AudBility, composto por questionário de autopercepção e tarefas auditivas. As tarefas incluíram localização sonora, fechamento auditivo, figura fundo, escuta dicótica, resolução temporal e ordenação temporal de frequência e duração. O programa foi reaplicado com um intervalo de 1 semana sob as mesmas condições de avaliação. O desempenho em cada tarefa foi apresentado a partir de dados de tendência central e dispersão e a confiabilidade a partir do Cálculo do Coeficiente intra-classe (CCI), com base no intervalo de confiança (IC) de 95%.

**Resultados:**

As análises demonstraram CCI positivo e significante para o questionário e tarefas auditivas, exceto fechamento auditivo, nas orelhas direita e esquerda e figura-fundo na orelha esquerda. O CCI médio do questionário foi de 0,742 e variou de -0,012 a 0,698 para as tarefas auditivas.

**Conclusão:**

Com base no resultado médio e no limite superior do IC, os achados demonstraram concordância entre os momentos de grau bom para o questionário, e de grau bom a moderado para cinco das sete variáveis auditivas analisadas (CCI>0,05 e < 0,9). Os achados do estudo de confiabilidade representam um parâmetro importante de validação do programa para a faixa etária estudada.

## INTRODUÇÃO

O processamento auditivo central (PAC) relaciona-se com a eficiência da percepção da informação auditiva pelo sistema nervoso auditivo central (SNAC) e a atividade neurobiológica subjacente, sendo um conjunto de habilidades específicas necessárias para a compreensão do que se escuta^(1)^. As habilidades relacionam-se com diferentes mecanismos auditivos, tais como a localização e lateralização do som, discriminação e reconhecimento de padrões auditivos, escuta dicótica, escuta em ambientes com sons competitivos ou sinal acústico degradado^(1)^.

Para avaliar as habilidades do PAC é fundamental compreender a relação entre o SNAC e sistema nervoso auditivo periférico (SNAP). O SNAP, constituído pelas orelhas externa, média e interna e nervo auditivo, é responsável por captar e transmitir os sinais auditivos para o SNAC, que irá interpretar a informação, sendo, portanto, processos distintos, porém interdependentes. A avaliação do PAC pode ser realizada tanto por meio de procedimentos eletrofisiológicos quanto por uma bateria de testes especiais comportamentais, possibilitando identificar a existência ou não de déficits no processamento neural dos estímulos auditivos e consequente comprometimento das habilidades auditivas avaliadas. Em caso de déficit, caracteriza-se o Transtorno do Processamento Auditivo Central (TPAC)^(1)^.

Estudos apontam que a disfunção auditiva periférica, acompanhada ou não de história de otite média secretora na primeira infância pode resultar em imaturidade no desenvolvimento de vias auditivas e habilidades auditivas centrais^(2,3)^. Além disso, alterações nas habilidades auditivas podem interferir no processo de aprendizagem e desenvolvimento da criança, devido ao fato de haver interdependência com as funções cognitivas superiores, tais como linguagem, memória e atenção^(4,5)^. A ocorrência do TPAC na criança em idade escolar pode coexistir com outros transtornos, como o Transtorno de Déficit de Atenção e Hiperatividade (TDAH), Transtornos do Desenvolvimento da Linguagem, Dislexia, entre outros^(6,7)^. Entretanto, existem manifestações específicas ao TPAC relacionadas aos déficits ao longo da via auditiva e que podem ocorrer também isoladamente, como a dificuldade em compreender a fala em ambientes ruidosos, baixa compreensão diante de situações com velocidade de fala aumentada, dificuldades em discriminar palavras ou sons similares e/ou seguir comandos auditivos complexos^(8)^. Essas queixas acarretam dificuldades que, na maioria dos casos, manifestam-se a partir de um baixo desempenho acadêmico^(9)^. Estudos demonstram associação entre a presença do TPAC e transtornos específicos de aprendizagem^(5)^, principalmente relacionado aos processos de aquisição da leitura e escrita e cálculos matemáticos, bem como déficits fonológicos^(10)^ e percepção da fala, incluindo aspectos suprassegmentais do discurso^(11)^.

A realização da triagem auditiva, composta por procedimentos que avaliem tanto o segmento periférico quanto central, possibilita a identificação precoce e intervenção imediata de alterações auditivas nessa população, minimizando os possíveis prejuízos escolares^(12)^. Nos últimos anos, o desenvolvimento de novas baterias de triagem das habilidades auditivas têm sido objeto de pesquisas^(13-15)^, as quais visam aliar as inúmeras possibilidades que o uso da tecnologia tem proporcionado na área da saúde com a necessidade de procedimentos que cumpram os requisitos necessários em uma triagem, considerando o fácil acesso e tempo de aplicação, diferentes mecanismos auditivos e, ao mesmo tempo, atividades interativas^(15)^.

A partir disso, destaca-se a necessidade de que dados de sensibilidade, especificidade e confiabilidade sejam estudados e disponibilizados, contribuindo para que novos procedimentos sejam validados. Nesse contexto, emergiu no cenário brasileiro a bateria de “AudBility”, um programa *online*, lúdico e interativo de triagem das habilidades auditivas, que vem sendo estudado e aperfeiçoado^(15)^. O programa é composto por tarefas auditivas e questionário de autopercepção para crianças, pais e professores. A fim de dar continuidade nos estudos do AudBility, e assim contribuir com a validação, o presente estudo teve como objetivo investigar a confiabilidade teste-reteste em um grupo de crianças com desenvolvimento típico e idades entre seis e sete anos.

## MÉTODO

Trata-se de um estudo de confiabilidade descritivo-analítico, quantitativo e de corte transversal, aprovado pelo Comitê de Ética em Pesquisa da Universidade Estadual de Campinas (Unicamp) sob o parecer Nº 1.561.422 e desenvolvido em parceria com uma Escola da Rede Pública Estadual de Ensino. Todas as crianças incluídas no estudo tiveram o Termo de Consentimento Livre e Esclarecido (TCLE) assinado pelos pais e/ou responsáveis e Termo de Assentimento assinado

### Seleção dos sujeitos e coleta de dados

Inicialmente, como parte do estudo de dados normativos do programa AudBility ao qual a presente pesquisa fez parte^(15)^, foram enviadas cartas-convite aos pais/responsáveis das crianças na faixa etária de seis a oito anos, do sexo masculino e feminino e falantes nativos do Português. Para este envio, foram excluídas crianças com diagnóstico já confirmado de perda auditiva periférica, além de alterações cognitivas/neurológicas que afetassem o desenvolvimento neuropsicomotor ou de linguagem que fossem de conhecimento da coordenação pedagógica, registros escolares e/ou professor da criança, condições que poderiam impedir a compreensão das tarefas a serem realizadas.

Ao todo, foram enviadas 203 cartas-convite. Destas, 157 (77%) aceitaram participar. Três crianças ainda foram excluídas devido aos critérios de seleção apresentados, totalizando 154 crianças que passaram pelos procedimentos de triagem periférica e AudBility. Das 154 crianças, 95 eram da faixa etária de oito anos e já haviam realizado a triagem há mais de dois meses, o que inviabilizou a inclusão desta idade na análise de confiabilidade. Além disso, para o presente estudo de confiabilidade, foram incluídas apenas as crianças com desempenho escolar adequado para a faixa etária de acordo com o professor responsável, incluindo ausência de retenção ou reforço escolar em período contraturno e que concordassem em participar da etapa de reteste. Sendo assim, a amostra final desta pesquisa foi constituída por 29 crianças na faixa etária de seis e sete anos, sendo 16 (55,17%) meninas e 13 (44,83%) meninos com média de idade 6,68 anos (±0,54). A Figura 1 ilustra o processo de seleção da amostra final.

**Figura 1:** Critérios de seleção da amostra final do estudo

Quanto aos procedimentos, previamente à aplicação da bateria AudBility, foi realizada meatoscopia com otoscópio da marca Hein e imitanciometria por meio do equipamento *Interacoustics* MT10, a fim de verificar as condições de orelha média. As crianças que apresentaram excesso de cerume foram encaminhadas para remoção e, posteriormente, reconvocadas para a coleta de dados. As crianças que apresentaram resultados alterados na triagem imitanciométrica, ou seja, curva timpanométrica do tipo B ou C e ausência de reflexos acústicos ipsilaterais, foram encaminhadas para avaliação audiológica completa, avaliação otorrinolaringológica e excluídas da análise de dados.

Após a triagem imitanciométrica, o programa AudBility foi aplicado em uma sala silenciosa disponibilizada pela escola, afastada das demais salas de aula e pátio. Em momentos de intervalo e mudança de turnos escolares, a aplicação foi interrompida pelo pesquisador. A bateria foi aplicada com o auxílio de uma fonoaudióloga com experiência no programa AudBility, sendo também uma das pesquisadoras signatárias desse estudo. Foi utilizado um computador *desktop* conectado à internet e fone de ouvido supra auricular com cancelamento de ruído marca Panasonic modelo RPHC720.

O AudBility é composto por um questionário de autopercepção das habilidades auditivas e as tarefas auditivas. Além disso, apresenta três módulos etários de apresentação das tarefas, sendo um direcionado para crianças de seis a oito anos; o segundo direcionado para crianças de nove a 12 anos desde que haja domínio da leitura e o terceiro para usuários acima de 13 anos. Nessa pesquisa foram estudados os dados coletados a partir da aplicação do módulo específico para a faixa etária de seis a oito anos. Nesse módulo, as atividades não exigem o domínio da leitura por parte do escolar e apresentam recursos pictográficos.

O questionário de autopercepção das habilidades- versão adaptada e inserida no programa^(16)^ contém doze perguntas a serem respondidas com relação a frequência com que a situação ocorre no dia a dia, com apoio pictográfico para as opções de resposta da criança, e forma de pontuação baseada na escala *Likert,* sendo: Sempre (1,0) Frequente (2,0) Algumas vezes (3,0) Raramente (4,0) Nunca (5,0). Cada pergunta é precedida por uma situação-exemplo para facilitar a compreensão. O pesquisador leu a tela de explicação do questionário no programa e, após garantir que a criança compreendeu, auxiliou na aplicação do questionário lendo as perguntas em voz alta e auxiliando na marcação da resposta na tela do computador. Ao final, o programa computa o escore por questão e calcula o escore total, que pode variar de 12 a 60 pontos. A descrição das 12 perguntas encontra-se no Quadro 1.

**Quadro 1:** Descrição do questionário de autopercepção estudado

**Questão**	**Situação-exemplo/Questão**
**Opções de resposta**	**Sempre/ Frequentemente/ Algumas vezes/ Raramente/ Nunca**
1	Você está em uma sala de aula ou em um ambiente em que tem pessoas conversando/ **Você tem dificuldade para escutar ou entender o que a professora está falando?**
2	A professora ou uma pessoa está falando muito rápido com você/ **Você tem dificuldade para entender o que a professora falou?**
3	A professora ou uma pessoa está dando instruções (explicações) faladas para você/ **Você tem dificuldade para seguir as instruções faladas?**
4	A professora ou uma pessoa está falando com você em um ambiente silencioso / **Você tem dificuldade para escutar e entender claramente as palavras sem trocar nenhuma letra?**
5	Quando a professora ou um amigo está falando com você/ **Você tem a sensação que às vezes você ouve bem e às vezes não?**
6	Você está em sala de aula ou no pátio da escola e alguém chama seu nome/ **Você tem dificuldade para perceber de onde vem o som?**
7	A professora ou uma pessoa está falando com você/ **Você pede para repetir o que foi falado?**
8	Você está na sala de aula/ **Você fica distraído com facilidade?**
9	No ano passado na escola/ **Você teve dificuldades para aprender?**
10	Você está fazendo uma atividade/ **Você tem dificuldade para ficar atento?**
11	Quando você está em sala de aula ou em casa/ **As pessoas falam que você é sonhador ou desatento?**
12	Quando você está na escola ou em casa/ **Você é desorganizado?**

Para as tarefas auditivas, o programa calibra automaticamente a saída do som e fones, sendo que o mixador do volume do computador foi posicionado em 50% e a criança questionada se estava em um nível confortável de audição. Caso necessário, o volume poderia ser ajustado.

O protocolo de triagem foi composto por sete tarefas auditivas, a saber: localização sonora, figura-fundo, fechamento auditivo, escuta dicótica com dígitos, resolução temporal e ordenação temporal de frequência e de duração. Como as tarefas de fechamento auditivo e figura-fundo foram analisadas por orelha, foram nove variáveis de análise. As tarefas são interativas e lúdicas e foram elaboradas com base nos testes comportamentais que fazem parte da bateria diagnóstica, porém diferentes parâmetros acústicos e em quantidade reduzida de estímulos em cada tarefa. O Quadro 2 apresenta a descrição de cada uma delas e a forma de pontuação.

**Quadro 2:** Descrição das habilidades e parâmetros de cada uma das sete tarefas que compuseram o protocolo de triagem.

**Tarefa AudBility**	**Parâmetros**
Localização Sonora	10 situações-alvo: direita, esquerda ou acima/ atrás
	10 apresentações – cada erro 10%
Integração Binaural (escuta dicótica com dígitos)	4 números apresentados concomitantemente (dois na orelha direita e dois na orelha esquerda).
	20 dígitos por orelha – cada erro 5% (resposta analisada com base no total de acertos obtidos OD+OE)
Figura Fundo (monoaural)	10 sequências por orelha em que a criança ouve uma história e de forma concomitante uma frase referente à figura e deve apontar a **figura**.
	Cada erro equivale a 10%
Fechamento Auditivo (monoaural)	10 sequencias por orelha em que a criança ouve uma palavra modificada acusticamente e deve reconhecer a palavra dentre as **figuras** apresentadas na tela.
	Cada erro equivale a 10%
Resolução Temporal	Estímulo (apito) simples de 1000Hz com intervalos entre eles - os gaps - com variações de 20ms,15 ms, 10ms, 6ms, 4ms e 0ms. Em cada apresentação, a criança ouve uma sequência de seis sons em que os gaps aparecem aleatoriamente é orientada a contar quantos consegue perceber/ouvir. Cada gap aparece 10 vezes.
	O Limiar considerado é o menor que ela percebe pelo menos 6 das 10 apresentações ou mais
Ordenação Temporal de Duração	10 sequências de três combinações entre tons puros de **800Hz/400ms** (LONGO-L) e **800Hz/ 200ms** (CURTO-C). Tempo de silêncio entre as sequências de **350**ms, (LLC, CCL, LCL, CLC, CLL e LCC).
	Cada erro equivale a 10%
Ordenação Temporal de Frequência	10 sequências de três combinações entre tons puros, sendo um estímulo grave (GROSSO-G) de **700Hz** e um estímulo agudo (FINO-F) de **1500 Hz** com duração de **350ms**, tais como GGF, FFG, FGF, GFG, GFF e FGG
	Cada erro equivale a 10%

**Legenda:** Hz = Hertz ; ms = milissegundos ; OD = orelha direita ; OE = orelha esquerda

Para cada tarefa, há uma tela de explicação e treino da atividade. A tarefa só foi iniciada após a garantia de que a criança compreendeu a atividade e o avaliador auxiliou a criança no momento da marcação da resposta na tela do computador. Ao final de cada tarefa, o programa apresenta uma tela parabenizando a criança pela execução e com a porcentagem de acertos e/ou limiar obtido. Essas informações também ficam armazenadas no programa e podem ser consultadas em uma tela com o resumo do desempenho da criança em todas as tarefas aplicadas, bem como escore do questionário (total e por questão). Nessa tela há também a informação do tempo total de aplicação da bateria e tempo por tarefa.

Após uma semana da aplicação do programa, os 29 escolares selecionados foram reconvocados para a etapa de RETESTE. Nessa etapa, o mesmo avaliador reaplicou os procedimentos da etapa teste, incluindo a meatoscopia, imitanciometria e o programa. Foram garantidas as mesmas condições da etapa TESTE no que diz respeito aos recursos materiais (sala, computador e fone), parâmetros de intensidade de apresentação dos estímulos e ordem das tarefas, instruções em cada tarefa e forma de aplicação/auxílio de marcação dos resultados. Nesse sentido, objetivou-se, na medida do possível, que os participantes fossem igualmente motivados e estivessem demonstrando o mesmo nível de atenção para realização das tarefas, considerando ambos os momentos de aplicação.

### Análise estatística

Para a análise de dados, o desempenho médio de cada uma das nove variáveis obtidas a partir da aplicação das tarefas foi calculado com base no percentual de acertos obtido ou limiar médio para a tarefa de resolução temporal. Esses dados foram apresentados a partir das medidas de tendência central e dispersão, assim como o escore médio obtido no questionário de autopercepção.

Para verificar a concordância ou discordância entre os momentos “TESTE” e” RETESTE”, ou seja, o grau de confiabilidade, foi realizado o Cálculo de Coeficiente intra-classe (CCI), com base em um modelo misto de dois fatores do tipo “concordância absoluta” a partir de medidas únicas^(17)^. Para análise, os valores negativos de CCI são interpretados como discordância entre os momentos, valores positivos são interpretados como concordância entre os momentos e valores nulos (zero) são interpretados como ausência de discordância ou concordância entre os momentos (acaso). Na análise do CCI, recomenda-se ainda que seja avaliado o nível de correlação, considerando não apenas o CCI médio mas também o intervalo de confiança de 95% adotado, a partir da seguinte classificação^(17)^: valores abaixo de 0,5 sugerem confiabilidade ruim, valores entre 0,5 a 0,75 confiabilidade moderada, valores entre 0,75 a 0,9 confiabilidade boa, valores acima de 0,9 confiabilidade excelente.

## RESULTADOS

A Tabela 1 apresenta as medidas de tendência central e de dispersão do resultado do questionário e desempenho médio de acertos nas tarefas da triagem de processamento auditivo obtidos por meio da aplicação do AudBility, de acordo com o momento de aplicação.

**Tabela 1:** Caracterização da amostra em relação ao resultado do questionário e tarefas do AudBility de acordo com o momento de aplicação (n=29)

**Variável**	**Momento**	**Média**	**DP**	**Mediana**	**Mín.**	**Máx.**
Questionário (escore)	Teste	45,72	7,46	46,00	30,00	56,00
		[42,87, 48,62]		[45,00, 49,50]		
	Reteste	45,66	6,91	46,00	30,00	57,00
		[43,15, 48,24]		[42,00, 50,00]		
Localização (%)	Teste	82,07	11,77	80,00	50,00	100
		[77,71, 86,40]		[80,00, 80,00]		
	Reteste	90,69	12,23	100	60,00	100
		[85,83, 94,84]		[100, 100]		
Dígitos – Geral (%)	Teste	82,84	8,47	85,00	67,50	97,50
		[79,67, 85,89]		[81,25, 85,00]		
	Reteste	88,71	8,25	92,50	67,50	100
		[85,69, 91,59]		[90,00, 92,50]		
Fechamento auditivo – OD (%)	Teste	89,66	12,10	90,00	60,00	100
		[85,00, 93,93]		[90,00, 90,00]		
	Reteste	97,59	5,11	100	80,00	100
		[95,67, 99,31]		[100, 100]		
Fechamento auditivo – OE (%)	Teste	87,24	16,23	90,00	30,00	100
		[80,39, 92,74]		[90,00, 90,00]		
	Reteste	96,21	6,22	100	80,00	100
		[93,79, 98,33]		[100, 100]		
Fígura Fundo Total - OD (%)	Teste	84,14	11,50	90,00	50,00	100
		[79,25, 88,13]		[90,00, 90,00]		
	Reteste	87,24	8,41	90,00	70,00	100
		[84,17, 90,34]		[90,00, 90,00]		
Fígura Fundo Total – OE %)	Teste	81,03	17,18	80,00	30,00	100
		[73,47, 87,14]		[80,00, 90,00]		
	Reteste	88,97	9,00	90,00	70,00	100
		[85,76, 92,00]		[90,00, 90,00]		
Resolução Temporal (Limiar- milissegundos)	Teste	5,38	4,21	4,00	4,00	20,00
		[4,10, 6,93]		[4,00, 4,00]		
	Reteste	4,62	2,98	4,00	4,00	20,00
		[4,00, 5,85]		[4,00, 4,00]		
Ordenação Temporal – Frequência - Total (%)	Teste	68,28	21,39	70,00	20,00	90,00
		[60,46, 75,12]		[60,00, 80,00]		
	Reteste	76,55	21,43	80,00	30,00	100
		[68,52, 83,45]		[80,00, 80,00]		
Ordenação Temporal – Duração – Total (%)	Teste	21,03	23,04	10,00	0,00	90,00
		[13,75, 29,66]		[10,00, 20,00]		
	Reteste	26,21	20,94	20,00	0,00	90,00
		[19,10, 33,79]		[10,00, 30,00]		

**Legenda:** DP: Desvio padrão; Mín.: Mínimo; Máx.: Máximo

A Tabela 2 apresenta a análise de confiabilidade teste-reteste para o questionário de autopercepção e tarefas da triagem de processamento auditivo do AudBility. As análises demonstraram CCI positivo, ou seja, concordante entre os momentos de aplicação, com significância estatística no questionário e em seis das nove variáveis das tarefas auditivas analisadas (p<0,05). Com base no resultado médio e no limite superior do intervalo de confiança (IC), os achados demonstraram concordância entre os momentos de grau bom para o questionário, e concordância que variou de grau moderado a bom para cinco das nove variáveis auditivas analisadas (CCI>0,05 e < 0,9), sendo moderado a bom para as tarefas de ordenação temporal de duração, resolução temporal, escuta dicótica com dígitos e figura fundo na orelha direita e moderado para a tarefa de ordenação temporal de frequência. A tarefa de localização sonora apresentou concordância que variou de ruim a moderada ao se considerar o limite superior do IC e observou-se concordância ruim para as tarefas de fechamento auditivo, nas orelhas direita e esquerda e figura fundo na orelha esquerda.

**Tabela 2:** Análise de confiabilidade teste-reteste para o questionário e variáveis auditivas referentes as tarefas auditivas triadas para a amostra estudada (n=29)

**Triagem**	**CCI**	**p**
Questionário	0,742	< 0,001*
	[0,519, 0,871]	
Localização	0,253	0,046*
	[-0,064, 0,543]	
Dígitos – Geral	0,558	< 0,001*
	[0,052, 0,803]	
Fechamento auditivo – OD	0,178	0,102
	[-0,107, 0,469]	
Fechamento auditivo – OE	0,095	0,268
	[-0,188, 0,400]	
Figura Fundo Total – OD	0,589	**< 0,001** *
	[0,296, 0,782]	
Figura Fundo Total – OE	-0,012	0,527
	[-0,317, 0,323]	
Resolução Temporal	0,698	< 0,001*
	[0,455, 0,845]	
Ordenação Temporal – Frequência - Total	0,510	0,001*
	[0,191, 0,733]	
Ordenação Temporal – Duração - Total	0,554	0,001*
	[0,249, 0,761]	

* Valor estatisticamente significante no nível de 5% (p ≤ 0,05)

**Legenda:** CCI – Coeficiente de correlação intraclasse

## DISCUSSÃO

Em um processo de validação, a confiabilidade do constructo está relacionada com a constância, precisão e homogeneidade dos resultados obtidos pelo método quando o indivíduo é avaliado mais de uma vez. Trata-se, portanto, de uma mensuração de extrema importância para que as medidas coletadas tenham significância. Existem diferentes critérios para o estudo da confiabilidade, sendo que um dos mais conhecidos e utilizados na área da saúde diz respeito a técnica do teste-reteste, ou seja, quando a mesma medida é aplicada duas vezes nos mesmos sujeitos e observa-se concordância entre os dois momentos^(18)^.

O uso dessa técnica pode ser afetado por fatores ambientais como avaliadores, características da amostra, tipo de instrumento e métodos de administração. Portanto, é imprescindível que as etapas sejam aplicadas sob as mesmas condições e analisadas de forma estatisticamente coerente^(19)^. O ideal é que os retestes, realizados com diferença de alguns dias a semanas do teste, tenham resultados semelhantes^(20)^, sendo que são descritos trabalhos na área do processamento auditivo com variações de até 60 dias entre os dois momentos de análise^(14,21,22)^. Em estudos de triagem, a taxa amostral varia de no mínimo cinco a 10% da amostra total triada^(13,14)^. No presente estudo, buscou-se respeitar tais preceitos, e as reavaliações ocorreram com uma semana de intervalo de tempo, foram realizadas pelo mesmo avaliador, sob as mesmas condições de avaliação. Quanto a taxa amostral, considerando as 154 crianças que passaram pela triagem, a amostra de 29 crianças representou 18,33% sendo, portanto, representativa.

Ao observarmos os dados descritivos apresentados na Tabela 1, tanto com relação ao escore obtido no questionário de autopercepção quanto nas tarefas auditivas, é possível verificar que a amostra apresentou um desempenho esperado para crianças com desenvolvimento típico, quando comparado com dados disponibilizados previamente, resultante da aplicação inicial do AudBility em crianças sem dificuldades escolares e desenvolvimento típico, porém com faixa etária maior^(15)^. No estudo citado, a faixa etária maior com resultado semelhante reforça o bom desempenho da amostra aqui estudada, sendo constituída por escolares mais novos. Além disso, a média de escore obtida no questionário acima de 45 pontos também condiz com o desempenho esperado apresentado em estudo realizado com o questionário SAB em sua versão traduzida^(23)^, reforçando a ausência do risco para a ocorrência do TPAC na amostra, ainda que selecionada com a limitação de um critério baseado na análise qualitativa do professor e registros escolares, não tendo sido feita uma avaliação formal do desempenho escolar.

Diferentes testes estatísticos podem ser utilizados para a análise de teste-reteste, sendo o Coeficiente de Correlação Intraclasse (CCI) um dos mais recomendados na literatura recente, por permitir de fato uma análise de concordância entre os momentos, além de diferentes medidas de correlação também serem utilizadas, tais como a Correlação Simples de *Pearson*, o Coeficiente de Correlação de *Spearman*, o teste *T Student* pareado ou o correspondente não paramétrico de *Wilcoxon* ^(17)^. Essa variabilidade de análises dificulta a comparação direta entre os diferentes achados, apesar de ser um tema frequente na área. Ao longo das últimas décadas, muitos trabalhos buscaram estudar a confiabilidade a partir de estudos de teste e reteste, incluindo as pesquisas relacionadas ao desenvolvimento de baterias de triagem ou testes do processamento auditivo^(13,14,19,24-27)^.

A boa confiabilidade teste-reteste do questionário de autopercepção aqui estudado reforça a importância da inclusão desse tipo de instrumento como avaliação complementar ao desempenho auditivo, em uma bateria de triagem auditiva escolar. Diversos autores ressaltam essa importância devido ao fato de o questionário ser uma forma simples e confiável, desde que elaborado com base em princípios psicométricos adequados e disponibilizados dados de validação, de demonstrar o impacto funcional do TPAC na vida cotidiana, sob um ponto de vista qualitativo^(23,28)^. O resultado também reforça os achados discutidos em publicação prévia em que o mesmo instrumento se mostrou eficaz para diferenciar um grupo de escolares quanto a variável do desempenho acadêmico^(16)^, além de ressaltar a necessidade das pesquisas de validação, incluindo não somente a confiabilidade teste-reteste mas também o estudo da sensibilidade e especificidade quanto ao ponto de corte para o risco e ocorrência do TPAC, a partir da correlação com testes diagnósticos^(13)^.

Apesar da variabilidade de estudos com discrepâncias com relação aos métodos de obtenção dos dados e de análise de resultados, é possível afirmar que os achados encontrados quanto a confiabilidade das tarefas auditivas estudadas, estão de acordo com outras pesquisas. A primeira versão da bateria SCAN-A *(Tests of Auditory Processing Disorders in adolescents and Adults)* ^(27)^, teve sua confiabilidade investigada a partir do teste e reteste em 38 participantes, com uma média de 40 dias entre os dois momentos de análise e os achados foram discutidos como insatisfatórios, tendo sido demonstradas fracas correlações na maioria das tarefas da bateria. Posteriormente, outros pesquisadores^(24)^ estudaram o SCAN-C, versão direcionada para crianças e encontraram melhora dos escores na segunda aplicação, sugerindo o efeito da prática. Ainda, uma versão revisada da bateria foi aplicada em uma amostra de 680 escolares, dos quais 145 (21,3% da amostra total) foram incluídos em uma análise teste e reteste com uma semana de intervalo entre as aplicações, tendo sido encontradas correlações baixas a moderadas, exceto para a habilidade de figura-fundo^(26)^, dados que corroboram com nossos achados. Já em outro estudo de validação que analisou uma bateria de triagem denominada STAP – *Screening Test for Auditory Processing* ^(14)^, 141 escolares foram triados pelo programa e, desses, 50 escolares (10% da amostra total) passaram pela etapa de reteste com dois meses de diferença entre as avaliações, e o coeficiente de correlação de *Pearson* demonstrou achados estatisticamente significantes que variaram de 0,82 a 0,93, sugerindo boa confiabilidade.

Mais recentemente, em um estudo inicial de validação de um programa computacional de triagem semelhante ao AudBility, denominado *Feather Squadron* ^(13)^, a viabilidade de aplicação do programa em diferentes faixas etárias foi estudada em conjunto com a análise de teste e reteste, realizada com uma semana de diferença entre os momentos de aplicação, envolvendo 5 mecanismos auditivos avaliados (localização sonora e lateralização, reconhecimento de padrões auditivos, aspectos temporais da audição, discriminação auditiva e performance auditiva com sons degradados). Com base no teste dos postos sinalizados de Wilcoxon, os autores verificaram o efeito da prática, a partir do melhor desempenho na segunda aplicação em três dos cinco mecanismos avaliados, bem como puderam verificar o mesmo efeito em três dos testes convencionais da bateria de avaliação comportamental do processamento auditivo, demonstrando correlação entre os procedimentos de triagem e diagnóstico, mas não em todos.

Sendo assim, os diferentes estudos discutidos apresentam baterias com o mínimo de concordância ou correlação moderada na maioria de tarefas que compõem protocolos de triagem do PAC, ainda que nem todos os testes demonstrem esse grau de correlação esperado^(13,14,24,27)^. Sendo assim, pode-se considerar que os achados do presente estudo apontam para a confiabilidade do programa estudado nessa faixa etária, contribuindo para sua validação.

Nossos achados não demonstraram resultados estatisticamente significantes nas tarefas de fechamento auditivo em ambas as orelhas e figura-fundo na orelha esquerda. O fechamento auditivo é a capacidade de compreensão de uma mensagem sonora incompleta ou distorcida, o que causa dificuldade em decodificar aspectos fonêmicos de um sinal de fala. Já a habilidade de figura-fundo consiste na interpretação de sentenças na presença de mensagem competitiva. Essas são habilidades auditivas fundamentais para a compreensão de fala, especialmente quando se trata do ambiente escolar^(29)^. Ao olharmos a descrição do desempenho médio de acertos nas tarefas, tanto para localização sonora cuja concordância foi baixa, quanto para as tarefas de fechamento e figura-fundo na orelha esquerda, é possível observar diferenças entre os momentos, com melhora do desempenho no reteste quando comparado com as demais tarefas com resultados positivos e com melhor grau de concordância, sugerindo um possível efeito da prática, ainda que uma análise estatística pareada dessa diferença não tenha sido realizada. Ainda assim, é possível considerar a hipótese de que essa diferença pode ter justificado a não concordância entre os momentos nessas tarefas.

Essa discussão e análise tornam-se relevantes, especialmente para futuros estudos que visem a aplicação da bateria AudBility com o objetivo de acompanhar o desenvolvimento maturacional das habilidades auditivas ou como instrumento de monitoramento da reabilitação do TPAC na criança. Autores ressaltam que o cálculo chamado de “diferença – D” que representa o grau de melhora ou piora entre os momentos deve ser considerado e descontado para se obter o real grau de melhora (ou piora) obtido pela criança ao longo do tempo em um processo de intervenção ou até mesmo para a adequada compreensão da sensibilidade do instrumento em mensurar o desenvolvimento neuromaturacional das habilidades auditivas ao longo do tempo^(19)^.

Alguns fatores devem ser discutidos e podem também subsidiar o levantamento de hipóteses a respeito das diferenças no desempenho entre os momentos, considerando as tarefas de baixa concordância. O fato de o desempenho escolar não ter sido avaliado por meio de um instrumento formal validado, mas sim a partir do relato do professor, justifica uma amostra mais heterogênea do ponto de vista dos processos de aprendizagem. Ainda que limitante, esse aspecto não inviabiliza o estudo de confiabilidade, visto que as análises são comparativas do sujeito com ele mesmo. Apesar do exposto, esse aspecto parece não justificar totalmente as discrepâncias encontradas, uma vez que o desempenho foi melhor no momento da segunda aplicação.

Outro aspecto a ser discutido é a influência que mecanismos cognitivos que independem do SNAC podem exercer sob o desempenho esperado do indivíduo submetido a avaliação do PAC, tais como memória e atenção^(30)^. Ainda que dentro do desenvolvimento típico, a ausência de avaliações formais, incluindo também um *screening* cognitivo e de linguagem, favorece uma amostra mais heterogênea, fator que também pode ser considerado como parte da justificativa para a discordância entre os momentos em algumas tarefas. Porém, trata-se de uma aplicação de triagem escolar, e um protocolo extenso de avaliações inviabilizaria a proposta. Além dos aspectos cognitivos, não é possível desconsiderar os fatores comportamentais ou de variabilidades intrínsecas de cada indivíduo, tais como sono, cansaço, estado de alerta, fome e grau de motivação para a realização das atividades. Do ponto de vista metodológico, ainda que garantidas as mesmas condições técnicas de aplicação do programa nos dois momentos, não é possível controlar todos os aspectos citados. Não necessariamente a criança foi convocada no mesmo horário daquele período escolar, podendo estar mais ou menos cansada entre uma semana e outra, por exemplo. Do ponto de vista motivacional, a familiaridade com o recurso tecnológico pode ser um fator a mais de motivação e engajamento para a realização das atividades, fator que também pode ter contribuído para o melhor desempenho no segundo momento.

Por fim, vale ressaltar que, de uma maneira geral, o CCI médio obtido nas tarefas com resultado positivo significante equivale a um grau de confiabilidade moderado, porém ao considerarmos a análise baseada no intervalo de confiança, essa classificação variou de ruim a bom, sendo ainda um dado de incerteza do ponto de vista estatístico. Conforme discutido, ainda que em concordância com outras baterias de triagem já validadas, esse achado justifica a necessidade de mais estudos, que avaliem uma amostra maior de escolares, de forma a estreitar o intervalo de confiança obtido, além de investigar as propriedades psicométricas das tarefas em outras faixas etárias e outras populações pediátricas, o que poderia elucidar o comportamento do programa em relação a esse aspecto da validação em diferentes populações.

## CONCLUSÃO

Considerando a faixa etária estudada e os resultados encontrados, o programa AudBility demonstrou boa concordância para o questionário entre os momentos aplicados, moderada concordância para as tarefas de ordenação temporal de frequencia e duração, resolução temporal, figura fundo na orelha direita e escuta dicótica com dígitos e baixa concordância, ainda que estatisticamente significante, para a tarefa de localização sonora. Os achados do estudo de confiabilidade representam um parâmetro importante de validação do programa.
